# TLR5 Risk-Associated Haplotype for Canine Inflammatory Bowel Disease Confers Hyper-Responsiveness to Flagellin

**DOI:** 10.1371/journal.pone.0030117

**Published:** 2012-01-18

**Authors:** Aarti Kathrani, Angela Holder, Brian Catchpole, Lorena Alvarez, Kenneth Simpson, Dirk Werling, Karin Allenspach

**Affiliations:** 1 Department of Veterinary Clinical Sciences, Royal Veterinary College, University of London, Hatfield, Hertfordshire, United Kingdom; 2 Department of Pathology and Infectious Diseases, Royal Veterinary College, University of London, Hatfield, Hertfordshire, United Kingdom; 3 Department of Clinical Sciences, Cornell University, College of Veterinary Medicine, Ithaca, New York, United States of America; University of London, St George's, United Kingdom

## Abstract

Single nucleotide polymorphisms (SNP) in the TLR5 gene have been associated with human inflammatory bowel disease (IBD) and animal models of this disease. We recently demonstrated a significant association between three non-synonymous SNPs in the canine TLR5 gene and IBD in German shepherd dogs (GSDs). However, so far, no direct link between these SNPs and a disturbance in TLR5 function was shown. In the present study, we determined the functional significance of the canine TLR5 SNPs by transfecting the identified risk-protective and risk-associated haplotype into human embryonic kidney cells (HEK) and assessed nuclear factor-kappa B (NF-κB) activation and CXCL8 production after stimulation. In addition, a whole blood assay for TLR5 activation was developed using blood derived from carrier dogs of either haplotype. There was a significant increase in NF-kB activity when cells transfected with the risk-associated TLR5 haplotype were stimulated with flagellin compared to the cells expressing the risk-protective TLR5 haplotype. This difference in NFkB activation correlated with CXCL8 expression in the supernatant measured by ELISA. Furthermore, whole blood taken from carrier dogs of the risk-associated TLR5 haplotype produced significantly more TNF after stimulation with flagellin compared to that taken from carriers of the risk-protective haplotype. Thus, we show for the first time a direct functional impact of the canine IBD risk-associated TLR5 haplotype, which results in hyper-responsiveness to flagellin compared to the IBD risk-protective TLR5 haplotype. Our data potentially suggest that similarly to human IBD and experimental models, TLR5 may also play a role in canine IBD. Blocking the hyper-responsive receptor found in susceptible dogs with IBD may alleviate the inappropriate inflammation seen in this disease.

## Introduction

The healthy gut is able to regulate inflammatory responses to commensal bacteria and food antigens whilst maintaining the capacity to respond to pathogens [1] [2] [3]. This distinction relies in part on appropriately functioning pattern recognition receptors (PRRs) such as Toll-like receptors (TLRs); which are ideally situated in intestinal epithelial cells and recognise microbe associated molecular patterns (MAMPs) [4]. In humans, the breakdown in this immunological tolerance to commensal and dietary antigens is thought to play an important role in the pathogenesis of inflammatory bowel disease (IBD) [2] and the same may also be true in dogs.

IBD is a chronic debilitating disease and occurs in both, humans and dogs. In dogs, it represents a group of disorders characterised by chronic gastrointestinal signs, with histological evidence of inflammation in the lamina propria of the small intestine, large intestine or both [5]. In humans, IBD consists of Crohn's disease (CD) and ulcerative colitis (UC) [6]. The histopathological changes seen in human IBD are different to the ones in canine IBD, in that extensive granulomas are the pathognomonic features of CD [7]. Although the exact aetiology of IBD in both species is unknown, it is considered a multi-factorial disease with the mucosal immune system, microbiota, genetics and environment all playing a role [8] [7] [6] [9]. Given the similarity of clinical signs, it is likely that canine and human IBD share similar aetiological factors as studies have already highlighted the importance of TLRs in IBD in both species. In human IBD, TLR 2, 4 and 6 have been shown to be upregulated in the intestinal mucosa of affected patients [10] [11] [12], whilst some studies have documented a down-regulation of TLR5 in the mucosa of mouse models and patients with UC [13] [14]. Similarly, we have recently shown an upregulation of TLR4 and a down-regulation of TLR5 at the mRNA level specifically in German shepherd dogs (GSDs) with IBD [15].

Moreover, we recently identified three non-synonymous single nucleotide polymorphisms (SNPs) present in the TLR5 exon (G22A, C100T and T1844C) that are significantly associated with IBD in GSDs [16]. One of these, G22A was found to be positively associated with IBD whereas the other two SNPs, C100T and T1844C were found to be significantly protective. GSDs carrying the TLR5 risk-associated haplotype (ACC) were five times more likely to develop IBD [16]. Furthermore, two of the non-synonymous TLR5 SNPs (C100T and T1844C) were also significantly protective for the development of IBD in 38 other canine breeds [17].

Genetic studies conducted in human IBD have shown that a polymorphism in the TLR5 gene (C1174T) results in a truncated form of TLR5 that is significantly protective for CD [18]. Therefore, as polymorphisms in TLR5 are associated with disease phenotypes in dogs and humans, this suggests that certain polymorphisms have functional consequences. Indeed, functional studies in both humans with IBD and animal models have confirmed that TLR5 plays a significant role in the aberrant inflammation seen in this disease [19]. The human truncated TLR5 has been shown to abrogate flagellin induced signalling [19]. Thus, a non-functional TLR5 receptor may be protective for CD. Moreover, studies in a murine model of IBD suggest that TLR5 signalling could play a protective role in IBD as TLR5 knockout mice develop spontaneous colitis [20].

As the canine genome exhibits vast linkage disequilibrium [21], it is possible that the SNPs found in the TLR5 gene that are significantly associated with canine IBD may be in linkage with another, possibly causative gene. Hence to confirm a primary role of TLR5 in canine IBD, functional studies would be needed. Therefore the aim of this study was to determine the potential functional significance of the canine TLR5 SNPs on the pathogenesis of IBD *in vitro* and *ex vivo*. Our results suggest that a hyper-responsive TLR5 receptor may play a role in the inappropriate inflammation seen in some cases of canine IBD.

## Results

### Confirmation of successful production of canine risk-protective (RP) and risk-associated (RA) TLR5-YFP by HEK 293 cells

In order to compare the functional response of the indentified polymorphisms, the TLR5 sequence of the IBD risk-protective haplotype (G22A haplotype GTT) and the IBD risk-associated haplotype (G22A haplotype ACC) [16] were cloned in to pcDNA3.1 plasmids containing YFP to monitor successful expression. To enhance the uniformity of the transformed population, cells stably expressing either form of TLR5-YFP were subsequently analysed and sorted for high expressing cells, resulting in a similar percentage of cells expressing the respective molecule.

### Confocal microscopy

**Figure 1:**
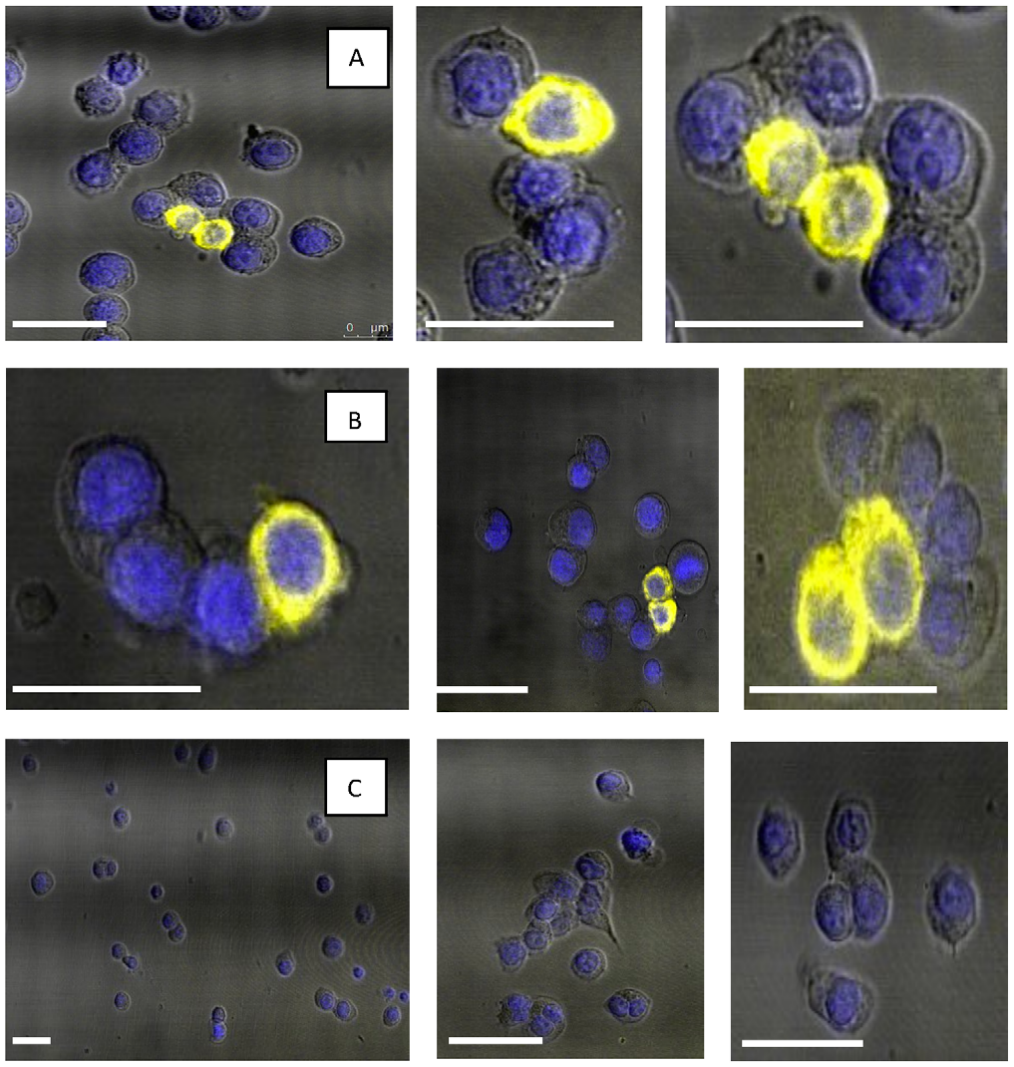
Confocal microscopic analysis of canineTLR5RP-YFP and canineTLR5RA-YFP. Transfected HEK 293 cells were cultured as described and analysed for fluorescence. Row A: Overlay of fluorescence of YFP conjugated to canineTLR5RP (yellow) and Hoechst stained nuclear fluorescence (blue); Row B: Overlay of fluorescence of YFP conjugated to canineTLR5RA (yellow) and Hoechst stained nuclear fluorescence (blue); Row C: Hoechst stained untransfected HEK 293 cell nuclear fluorescence (blue). Bar, 50 µm.

As flow cytometric analysis does not allow for complete discrimination of surface versus intracellular expression of canineTLR5-YFP, cells were analysed additionally by confocal microscopy. In agreement with the FACS data, YFP expression was detected in a similar percentage of HEK 293 cells transfected with both caTLR5 variants. YFP expression concentrated in a ring-like shape at the periphery of the cell as well as dispersed throughout the cytoplasm, suggesting presence of both canineTLR5RP-YFP and canineTLR5RA-YFP predominantly at the cell surface. No YFP signal was seen in control HEK 293 cells (Figure 1).

**Figure 2:**
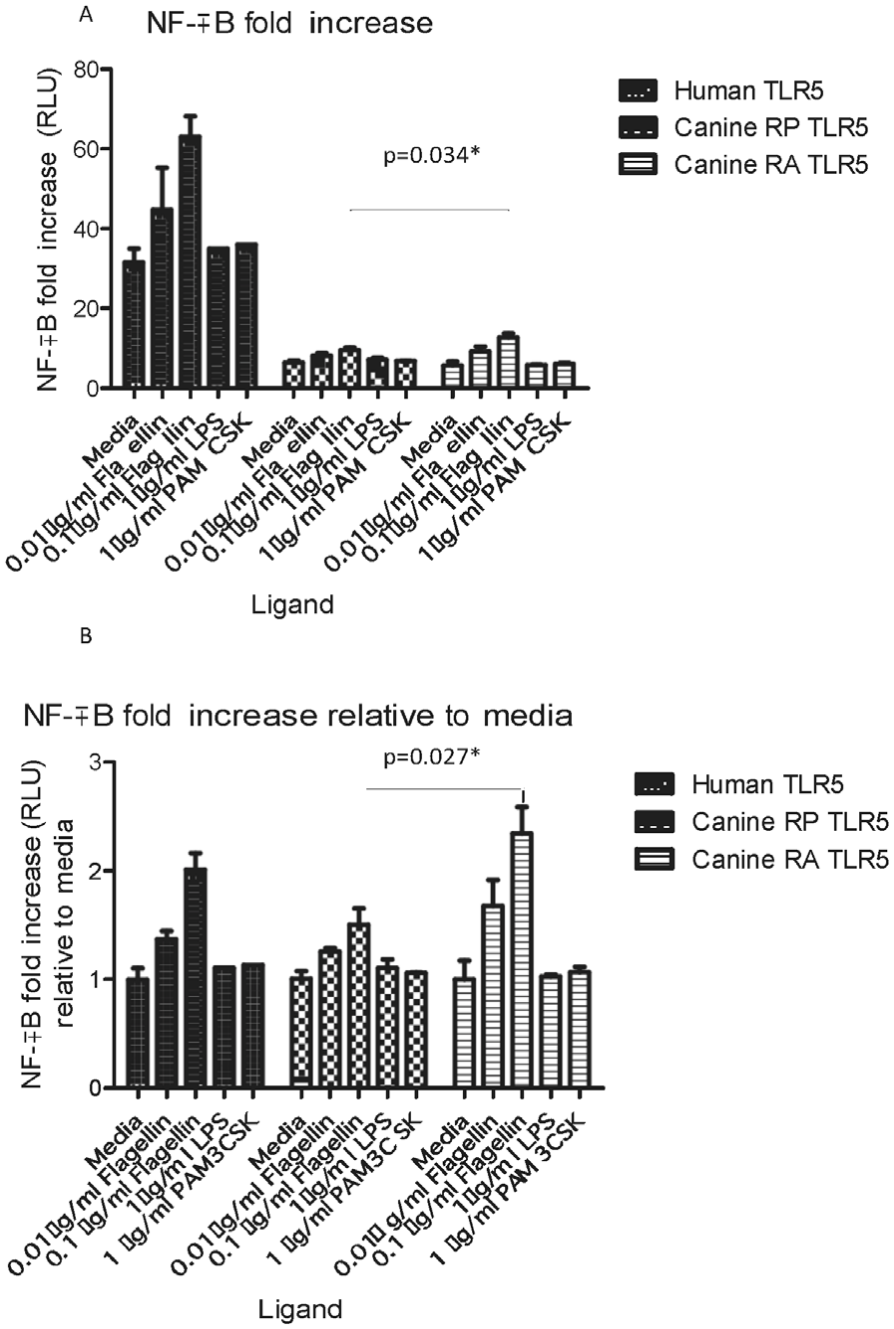
Activation of NF-κB in stimulated HEK 293 cells stably transfected with human TLR5, canineTLR5RP-YFP or canineTLR5RA-YFP. Transfected human TLR5, canineTLR5RP-YFP and canineTLR5RA-YFP HEK 293 cells were incubated with flagellin, PAM_3_CSK_4_, LPS or media for 24 hours before production of NF-κB was analysed by a luciferase reporter gene assay. The results displayed are the mean values of four independent experiments each with three repeats under the same conditions, with error bars illustrating standard error of the mean. Data are presented either as relative luciferase units (RLU) (A) or converted into fold-increase by comparing with RLU values obtained for unstimulated cells with media (B). There was a significant increase in the baseline and fold-increase in RLU values in canineTLR5RA-YFP compared to canineTLR5RP-YFP when the cells were stimulated with 0.1 µg/ml of flagellin (p = 0.034).

Having established expression of both caTLR5 constructs, we next assessed their functionality in response to different TLR ligands. To do so, HEK293 cells transfected with either human TLR5, canine TLR5RP or canine TLR5RA were stimulated with recombinant FliC (rFliC), LPS or PAM_3_CSK_4_, and the results compared to unstimulated cells. Whereas absolute values were in general higher in HEK293 cells transfected with human TLR5 (Figure 2), all responses showed a very similar response pattern when data were expressed relative to the medium-control (Figure 2). Interestingly, stimulation of RA-caTLR5 compared to RP-caTLR5 resulted in a significantly increased NF-kB activation when cells were stimulated with the higher concentration of rFliC (0.1 ug/ml) (p<0.05), but not with the lower dose (0.01 ug/ml) (p = 0.47) (Figure 2). In contrast, none of the other TLR-ligands tested resulted in an increase in NF-kB activation (Figure 2).

### CXCL8 production is increased after stimulation of RA-caTLR5

**Figure 3:**
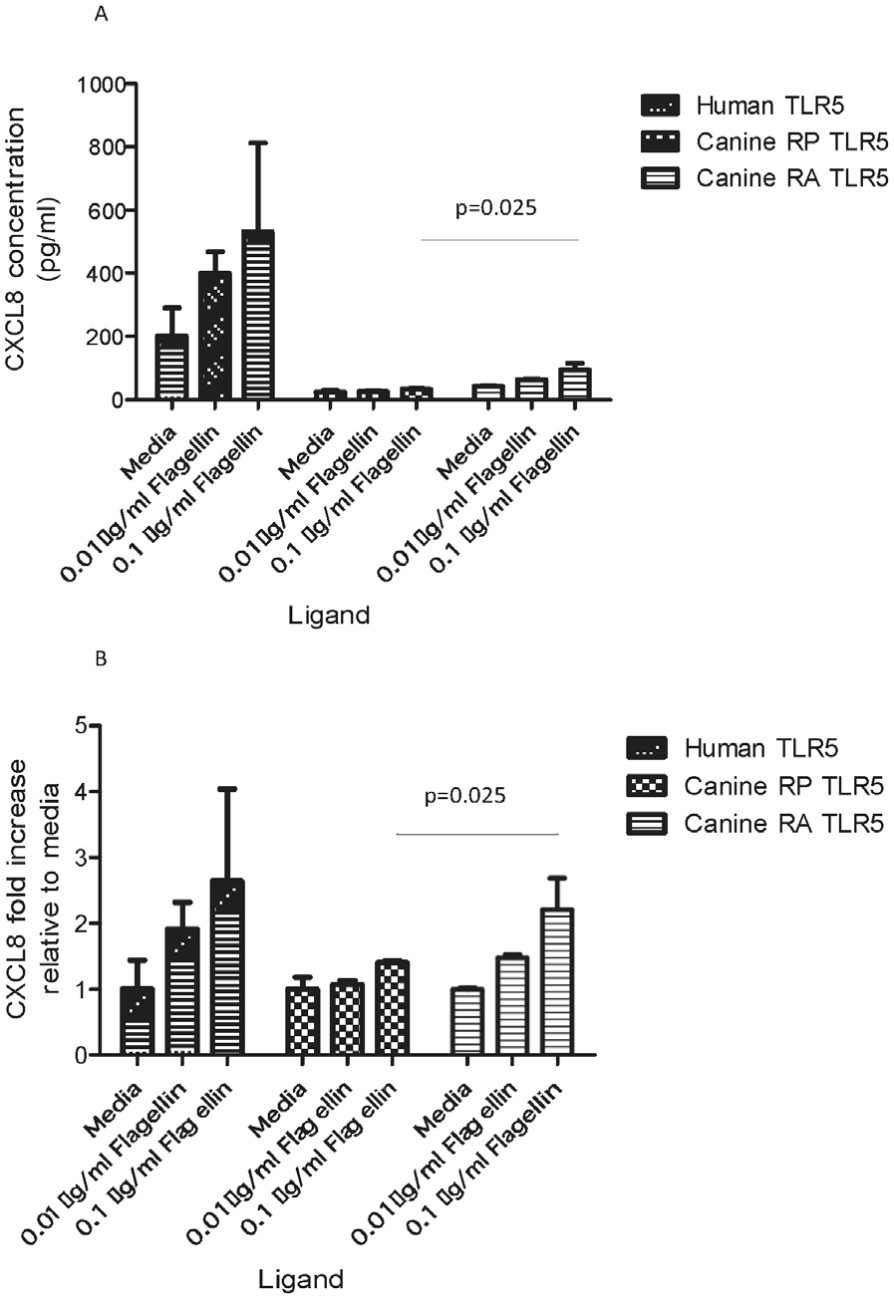
Activation of CXCL8 secretion in stimulated HEK 293 cells stably transfected with human TLR5, canineTLR5RP-YFP or canineTLR5RA-YFP. Transfected human TLR5, canineTLR5RP-YFP and canineTLR5RA-YFP HEK 293 cells were incubated with flagellin or media for 24 hours before production of CXCL8 was analysed by an ELISA. The results displayed are the mean values of one experiment with three repeats under the same conditions, with error bars illustrating standard error of the mean. Data are presented either as concentration (pg/ml) (A) or converted into fold-increase by comparing to the concentration obtained for unstimulated cells with media (B). The CXCL8 production was significantly higher in canineTLR5RA-YFP cells than in canineTLR5RP-YFP cells when stimulated with the higher flagellin concentration (0.1 µg/ml) (p = 0.025, one-tailed).

As stimulation of RA-caTLR5 did lead to an increased NFkB activation, we next tested whether this was associated with an increase in protein production. Thus, CXCL8 production in supernatants taken from one of the above experiments was analysed by ELISA. Data are presented as either total CXCL8 concentration (Figure 3), or as fold-increase determined relative to unstimulated cells (Figure 3). Similar to the changes seen with regards to NF-κB activation, CXCL8 production was significantly increased in HEK293 cells transfected with RA-caTLR5 compared to RP-caTLR5 when using the higher rFliC concentration (0.1 ug/ml) (p<0.05), but not with the lower rFliC concentration (0.01 µg/ml) (p = 0.47).

### Carriers of the RA-TLR5 haplotype show increased TNF production in response to flagellin

**Figure 4:**
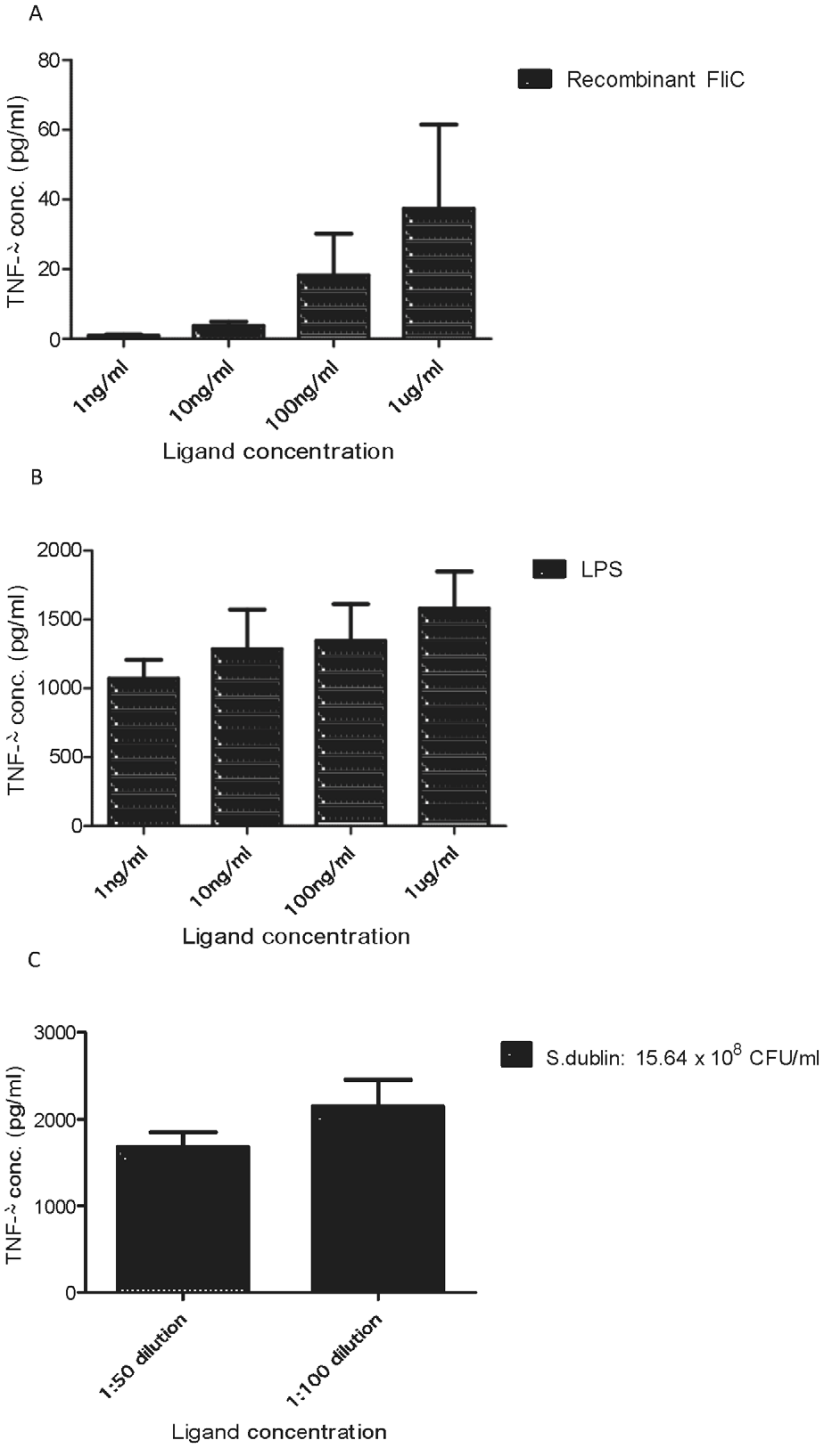
Activation of TNFα secretion in whole blood stimulation assays with flagellin, LPS or *Salmonella dublin*. Whole blood was diluted with twice the volume of RPMI and stimulated with flagellin, LPS or heat-inactivated *Salmonella dublin* at different concentrations for 24 hours before production of TNFα was analysed by an ELISA. The results displayed are the mean values obtained from four healthy blood donor dogs (2 Labradors, 1 Greyhound and 1 Great Dane) each with two repeats under the same conditions, with error bars illustrating standard error of the mean.

To assess whether the differences seen between the two TLR5 genotypes can be verified by means of an *ex-vivo* approach, a whole blood assay was developed. Having established the functionality of the whole blood assay with regards to different PRR ligands and concentrations in control dogs (Figure 4), GSDs for which TLR5 genotype information was available were recruited onto the study. Residual blood from the collection of haematology and serum biochemistry profiles was used in the whole blood assay as described in materials and methods.

A total number of 23 GSDs carrying the RP haplotype and 5 GSDs carrying the RA haplotype were recruited (table 1 and 2).

**Table 1:** Phenotype of German shepherd dogs carrying the risk-protective TLR5 haplotype which were recruited onto the whole blood stimulation assay study.

TLR5 Haplotype	Age at sampling	Sex	Diagnosis	Treatment of IBD at time of sampling
GGTTTT	4years	MN	Healthy	n/a
GGTTTT	5years	FE	Healthy	n/a
GGTTTT	7years	FE	Healthy	n/a
GGTTTT	7years	ME	Healthy	n/a
GGTTTT	8 years	MN	Healthy	n/a
GGTTTT	9 years	FN	Healthy	n/a
GGTTTT	8 years	MN	Healthy	n/a
GGTTTT	6 years	MN	UTI	n/a
GGTTTT	10 years	FE	Hemangiosarcoma	n/a
GGTTTT	7 years		Hemangiosarcoma	n/a
GGTTTT	6 years		Skin mass	n/a
GGTTTT	3 years		Lameness	n/a
GGTTTT	12 years		Myelopathy	n/a
GGTTTT	1 year		Lameness	n/a
GGTTTT	7 years		GI foreign body	n/a
GGTTTT	8 years		Lymphoma	n/a
GGTTTT	5 years		Otitis	n/a
GGTTTT	6 years		IBD	Diet
GGTTTT	5years	ME	IBD	Diet
GGTTTT	4years	FN	IBD	Diet
GGTTTT	6years	ME	IBD	Diet
GGTTTT	4years	FN	IBD	Diet
GGTTTT	4 years	MN	IBD	Corticosteroids

GSDs = German shepherd dogs, m = male, f = female, n = neutered, e = entire, IBD = Inflammatory Bowel Disease, n/a = not applicable.

**Table 2:** Phenotype of German shepherd dogs carrying the risk-associated TLR5 haplotype which were recruited onto the whole blood stimulation assay study.

TLR5 haplotype	Age at sampling	Sex	Diagnosis	Treatment of IBD at time of sampling
GGAGCT	7 years	FN	IBD	Diet
GGGGTT	6 years	FE	IBD	Diet
AGAGCT	4 years	FN	IBD	Diet
AACTCT	7 years	FN	IBD	Diet
AGCTCT	3 years	MN	Lameness	n/a

GSDs = German shepherd dogs, m = male, f = female, n = neutered, e = entire, IBD = Inflammatory Bowel Disease, n/a = not applicable.

**Figure 5:**
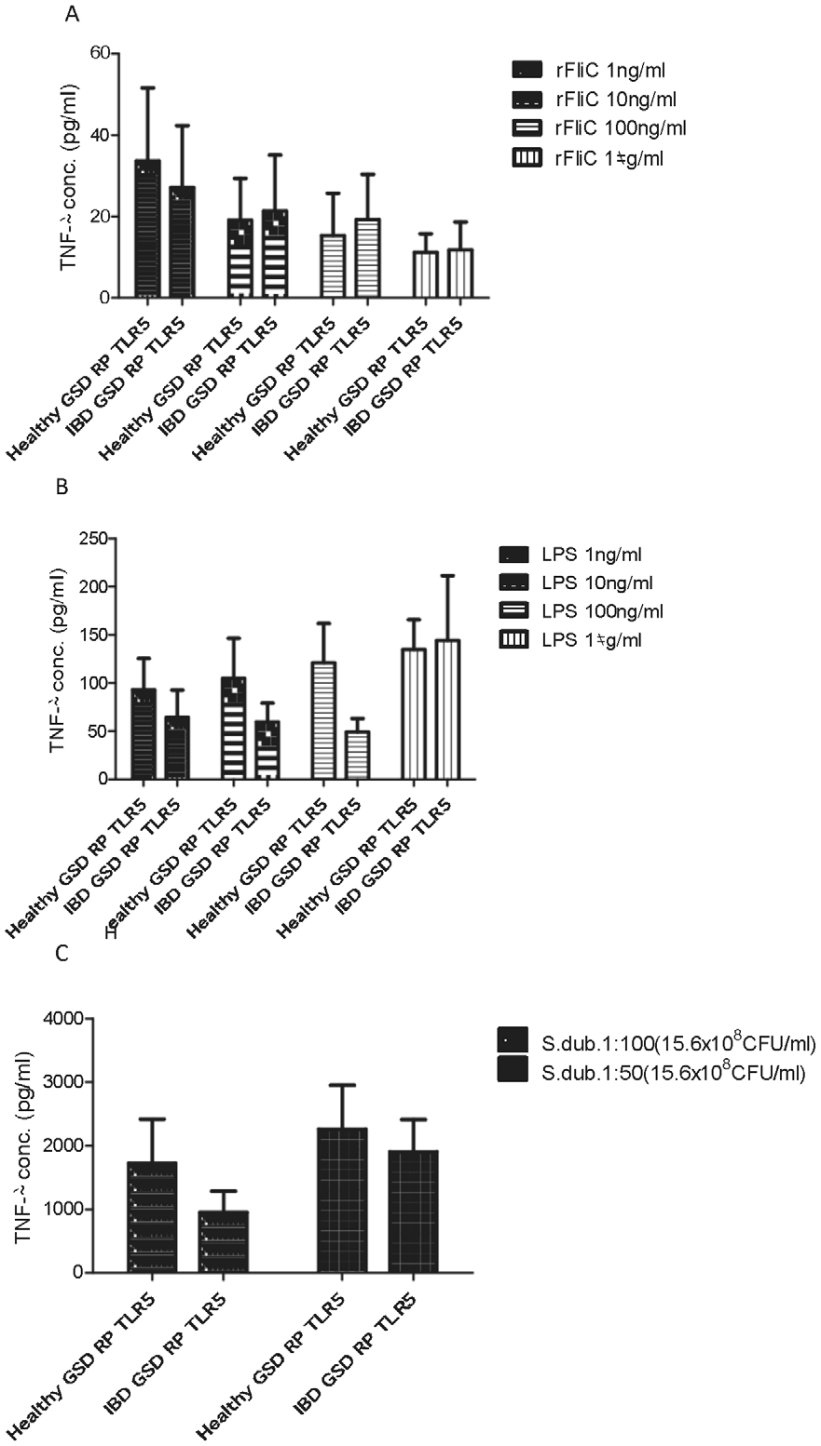
TNFα secretion in whole blood stimulation assays with flagellin, LPS or heat-inactivated *Salmonella dublin* in healthy GSDs and those with IBD carrying the risk-protective TLR5 haplotype. Whole blood was diluted with twice the volume of RPMI and stimulated with flagellin, LPS or heat-inactivated *Salmonella dublin* at different concentrations for 24 hours before production of TNFα was analysed by an ELISA. The results displayed are the mean values obtained from five dogs, with error bars illustrating standard error of the mean. There was no significant difference in TNFα response to flagellin, LPS or *Salmonella dublin* between healthy GSDs and those with IBD all carrying the risk-protective TLR5 haplotype at any of the concentrations tested (p>0.05).

Comparing 5 GSDs each carrying the RP TLR5 haplotype but with different phenotype (IBD vs healthy), no significant differences in TNF production were seen in response to the stimulation, independent of disease status. Increasing amounts of rFliC led to a reduced production of TNF (Figure 5). In contrast, there was a tendency to an increased production of TNF in response to increasing amounts of LPS (Figure 5), and hardly any differences were seen when using the different *S. dublin* dilutions (stock concentration: 15.64×10^8^ CFU/ml).

**Figure 6:**
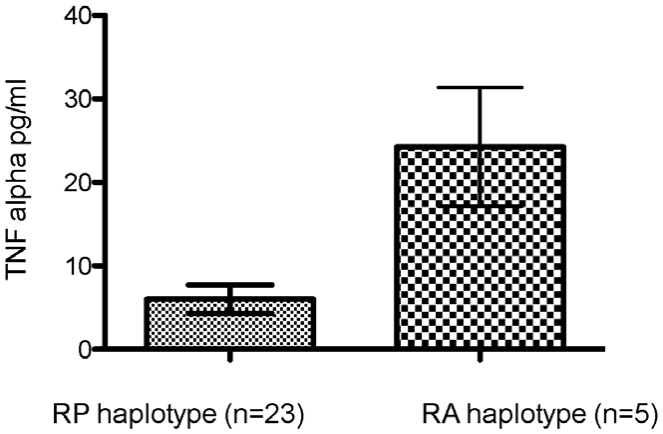
Stimulation of TNF-α secretion in whole blood stimulation assays with flagellin in GSDs with IBD carrying either the risk-protective TLR5 haplotype (RP) or risk-associated polymorphisms in TLR5 (RA). Whole blood was diluted with twice the volume of RPMI and stimulated with 1 ug/ml flagellin (FliC) for 24 hours before production of TNF-α was analyzed by an ELISA. All assays were performed in duplicates. The boxes displayed represent ranges with lines representing the median values obtained from 23 GSDs with IBD carrying the RP TLR5 haplotype and from 5 GSDs with IBD carrying the risk-associated haplotype in their TLR5 gene (3 different haplotypes: AGAGCT, GGGGTT and GGAGCT).

Comparing the results obtained for GSD with the RP (n = 23) or RA (n = 5) TLR5 haplotype independent of disease status showed that GSDs carrying the RA TLR5 produced significantly more TNF-α in the supernatant compared to GSDs carrying the RP TLR5 after stimulation with flagellin (median TNF-α production in RP dogs: 3.3 pg/ml, range 0–27.4; median TNF-α production in RA dogs: 16.7 pg/ml, range 8.9–47.7; Mann Whitney U p = 0.006) (figure 6).

Expression of TLR4 and TLR5 has been reported for human peripheral blood T lymphocytes, monocytes, neutrophils and eosinophils [22] [23] [24] [25] [26]. Although, in dogs it has been shown that blood-derived monocytes express TLR4 and TLR5 and stimulation with LPS and flagellin results in TNFα production [27], data on TLR expression on eosinophils, neutrophils and lymphocytes are lacking in this species. Nevertheless, to ensure that any significant differences seen in TNFα production were not due to significant differences between the concentrations of leucocyte subsets, statistical analysis was carried out on the haematology results obtained from each dog. There were no significant differences in the numbers of neutrophils, monocytes or eosinophils between the group of healthy GSD and the group of GSD with IBD (p>0.05; data not shown). However, there was a borderline significantly greater concentration of lymphocytes in the healthy GSD group compared to the IBD group (p = 0.05). In addition, there were no significant differences in the concentrations of neutrophils, lymphocytes, monocytes or eosinophils between the IBD GSD group with RP TLR5 haplotype and the IBD GSD group with RA TLR5 polymorphisms (p>0.05; data not shown). All parameters for the serum biochemistry profile were within the reference range for all dogs.

## Discussion

As initial association studies suggested a significant effect of canine TLR5 SNPs with the occurrence of IBD in GSDs [16], we investigated whether these SNPs resulted in functional differences of the expressed protein constructs. The results of the *in vitro* analysis using both TLR5 haplotypes expressed in HEK cells described in the present study clearly demonstrate that the TLR5 risk-associated haplotype (ACC) is significantly hyper-responsive to flagellin compared to the TLR5 risk-protective haplotype (GTT). In addition, we were able to show that this hyper-responsiveness also exists when whole blood is stimulated with the appropriate ligands. The observed hyper-responsiveness to flagellin could in part be responsible for the inappropriate inflammation seen in dogs with IBD carrying this haplotype of the TLR5 gene.

The importance of defective TLR5 haplotypes for the pathogenesis of IBD has been demonstrated before in human patients and animal models [18], [20]. In human subjects, a stop codon in TLR5 which abrogates flagellin signalling is associated with protection against CD in a Jewish cohort [18], suggesting that abolishing flagellin signalling may help prevent IBD. In addition, subjects with this TLR5 genotype produced significantly lower concentrations of pro-inflammatory cytokines in intestinal lesions compared to individuals carrying the wild-type genotype [19]. Moreover, in recent publications we were able to show a genetic association between specific TLR5 haplotypes and the development of IBD in GSDs as well as in 38 other canine breeds [17] [16].

The data of the present study suggest that dogs carrying the risk-associated TLR5 haplotype are hyper-responsive to flagellin. Thus, it could be speculated that this hyper-responsiveness plays a significant role in the pathogenesis of canine IBD. Therefore, blocking the excess response to flagellin in susceptible dogs could potentially help abolish the inappropriate inflammation seen in canine IBD. However, under physiological conditions, TLR5 may indeed exert a beneficial effect in the gut. Sensing of flagellin/flagellated bacteria by TLR5 expressed in the intestinal epithelia has been shown to enhance the expression of bacteriocidal and barrier-enhancing mediators [28] [29] [30] [31], to reduce epithelial cell apoptosis, to limit disease severity during enteric infection [31], and to contribute to epithelial repair [31]. This suggests that epithelia are able to re-establish homeostasis in an autonomous non-inflammatory manner by sensing flagellin directly via TLR5 [31]. Therefore TLR5 also plays a beneficial role in the gut and blocking such signalling entirely may impair important defence mechanisms. Indeed, it has been shown that TLR5 knockout mice have more severe gut pathology and develop spontaneous colitis [20].

However, the presence of the RA haplotype alone may not entirely explain the differences seen in clinical signs. Interestingly, a recent report suggests that whilst epithelial NF-κB activation may be protective, immune cell-derived NF-κB activation drives colitis [32]. Furthermore, TLR5 ligation by flagellin converted tolerogenic DCs into activating APC, producing IL12 but not IL10, and promoting differentiation of naïve T cells into TH1 cells [33]. Similarly, murine DCs present in the intestinal lamina propria display high levels of TLR5-mediated production of IL12, but not IL10 when stimulated with flagellin [34]. Thus, epithelial TLR5 activation may exert a protective role in the gut, whereas breach of this barrier during inflammatory conditions such as seen in IBD could lead to an exaggerated response to flagellin, similar as seen in dextran-sodium sulphate induced colitis in mice [35]. It could therefore be speculated that in dogs with IBD, a defective or leaky intestinal barrier may allow increased contact between flagellin and TLR5 on immune cells, therefore increasing mucosal inflammation in dogs carrying the RA haplotype. In addition, this could explain why those dogs carrying the TLR5 RP haplotype produce significantly less CXCL8 and are therefore less susceptible to the development of chronic intestinal inflammation such as in IBD.

In conclusion, TNF production in response to flagellin stimulation was significantly higher in the group of IBD dogs carrying TLR5 RA polymorphisms compared to the IBD group carrying RP TLR5. The data from the whole-blood assay therefore support those obtained using TLR5-expressing HEK293 cells with regards to NF-κB activity and CXCL8 production.

As the results of one study indicated that TLR5 activation systemically and within the mucosal surface may be controlled by different mechanisms [36], further studies should aim to confirm that risk-associated polymorphisms in canine TLR5 result in hyper-responsive signalling to flagellin within the intestinal mucosa.

## Materials and Methods

### Cloning of the risk-protective and risk-associated TLR5 haplotype

Primers were designed to incorporate a 5′EcoR1- and a 3′ HinD3 restriction site, a 5′ KOSAK sequence as well as the removal of the internal stop-codon for both, the risk-protective and risk-associated sequence (Table 3). The PCR reactions were carried out with genomic DNA from a healthy GSD carrying the risk-protective TLR5 haplotype (GTT) and from a GSD with IBD carrying the risk-associated TLR5 haplotype (ACC) using the Easy A 2× master mix (Stratagene, California, USA) by heating to 95°C for 10 min, followed by 35 cycles of 95°C for 1 min, 62°C for 1 min and 72°C for 3 min and a final extension at 72°C for 7 min. Resulting PCR products were analysed on a 1% agarose/safeview nucleic acid stain gel (NBS Biologicals, Huntingdon, UK) using 6× orange loading dye (Fermentas) in 1× Tris-Borate EDTA (TBE) buffer. Bands of the correct size were excised and DNA extracted using the Qiagen mini-elute extraction kit, according to manufacturer's instructions. Purified cDNA was subsequently ligated into pSC-A-amp/kan (Agilent Technologies, UK) and used to transform SoloPack competent cells (Agilent Technologies, UK) according to the manufacturer's instructions. Subsequently, colonies were picked, grown overnight in LB liquid Ampicillin (50 µg ml^−1^) broth (Fermentas) and plasmid was extracted using GenElute plasmid Miniprep Kit (Sigma) according to the manufacturer's instructions before sequencing (Geneservice, Cambridge). To monitor expression in HEK293 cells, the TLR5 constructs were subsequently subcloned by restriction-enzyme digest into pcDNA3.1-YFP. One Shot TOP10 competent cells (Invitrogen) were transformed according to the manufacturer's instructions and plasmid extracted from cells using the method described above. The resulting plasmid (caTLR5-YFP) was sequenced for correct reading frames (Geneservice, Cambridge).

**Table 3:** Primer sequence used for PCR amplification of full-length canine TLR5 to incorporate EcoR1 restriction site and KOZAK sequence to 5′ end and to remove stop codon and add HIND3 restriction site to 3′ end.

TLR5 restriction site primer FORWARD sequence	5′-AAGCTTACCATGGGCCGTCAGCTGG-3′
TLR5 restriction site primer REVERSE sequence	5′-AGCCAGCTTGGCGACGGCC-3′

### Expression of canine TLR5-YFP in HEK293 cells

HEK293 cells (Invivogen, UK) were cultured at 37°C, 5% CO2 in Dulbecco's Modified Eagle's Medium (DMEM, Gibco, UK) with 10% foetal calf serum (FCS, γ-irradiated and filtered at 0.22 µM, Sigma-Aldrich,UK) and Penicillin-Streptomycin (100 units ml^−1^ and 0.1 mg ml^−1^ respectively) for untransfected cells. After transfection with either canine risk-protective or risk-associated TLR5-YFP using Lipofectamine 2000, stable lines were created by subsequent selection in geneticin (1000 µg ml^−1^). After passaging, transfected and un-transfected HEK293 cells were analysed for YFP-expression using a FACSAria (BD Biosciences, Oxford, UK). Human TLR5 transfected HEK cells (Invivogen) were used as positive control and as a comparison to the canine system in experiments. To increase homogeneity of expression, caTLR5-YFP expressing HEK293 cells were sorted on high expressing cells using the FACSAria, and subsequently analysed by confocal microscopy. To do so, cells were seeded on to the glass circle of 35 mm poly-d-lysine coated Glass Bottom Culture Dishes (MatTek). Dishes were incubated at 37°C and 5% CO_2_ for 4 hours for the cells to adhere after which 2 ml of DMEM was added to dishes which were then incubated at 37°C and 5% CO_2_ for 24 hours. After incubation media was removed and cells were fixed subsequently by adding 200 µl of 4% paraformaldehyde and incubating at room temperature for 15 mins. Cells were then carefully washed twice with sterile PBS. Four micrograms per ml of Hoechst stain in PBS with 0.05% saponin (Sigma-Aldrich) was added to cells (100 µl per plate) and left to incubate for 10 mins at room temperature. Cells were then washed twice with molecular biology water (Sigma) and 1 drop of vectorshield (Vector Laboratories) was added to the glass area of each dish. A cover slip was applied and Eukitt was used to seal the cover slip to the dish. Dishes were stored at 4°C and away from light until being analysed using a Leica SP5 RS Confocal Microscope and LSM 510 Browser software.

### NF-κB-luciferase Transient Transfection

To assess the effect of caTLR5 SNPs on the ability of the receptor to confer an intracellular signal, Luciferase (luc)-tagged NF-κB was transfected into the transfected HEK293 cells, along with HSV-thymidine kinase promoter (pRL-TK) renilla as a transfection control. Human TLR5 transfected HEK293, risk-protective and risk-associated caTLR5 HEK293 cells were plated into a 24-well plate at 5×10^5^ cells in 1000 µl serum- and antibiotic-free DMEM per well and incubated at 37°C 5% CO_2_ for 24 hours. Thereafter, 0.5 µl Lipofectamine (Invitrogen, UK) was added to 12 µl DMEM to make a final volume of 12.5 µl, added to each well and incubated at RT for 5 mins. 100 ng NF-κB-luc plasmid and 4 ng pRL-TK renilla plasmid were mixed together in a final volume of 12.5 µl DMEM and added to the Lipofectamine/DMEM solution, which was then incubated at RT for 20 mins. The solution was then topped up with 75 µl DMEM and the resulting 100 µl solution added to the well. The cells were incubated at 37°C, 5% CO_2_, and 400 µl of the desired ligands (0.1 µg/ml recombinant FliC, 0.01 µg/ml recombinant FliC, 1 µg/ml LPS (Invivogen) or 1 µg/ml PAM_3_CSK_4_ (all Invivogen)) were added to the plate 24 hours later. After 24 hours stimulation, NF-κB-luc and pRL-TK renilla levels were analysed on a MicroLumat Plus LB96V machine (Berthold Technologies) using the Dual-Luciferase Reporter Assay System (Promega, UK), according to the manufacturer's protocol. Mean luciferase values were recorded and tabulated using WinGlow software (v.1.25.00003, Bethold Technologies). All assays were performed in triplicate.

### CXCL8 ELISA

To analyse whether the identified SNPs in caTLR5 do not only have a potential effect on transcription factor activity, but also on chemokine production, the R&D Systems Quantikine Human IL-8 Immunoassay was used to test the supernatant for CXCL8 of stimulated HEK cells according to the manufacturer's instructions. Optical density (OD) was measured at 450 nm with a 610 nm wavelength correction using a Bio-Tek EL8088 Channel Absorbance Reader (Fischer Scientific) within 30 minutes and the data was analysed using Gen5 Data Analysis Software (Biotek, Fisher Scientific). All assays were performed in triplicates.

### Whole Blood Stimulation Assay for TNFα

In a last set of experiments, we assessed whether similar effects as seen in the *in-vitro* assays could be seen in an *ex-vivo* approach. To do so, a whole blood stimulation assay was developed. Six millilitres of venous blood was collected from GSD carrying risk-associated and risk-protective TLR5 polymorphisms. All dogs had been referred to the RVC, either for assessment of various illnesses or for the purpose of a health screen (see table 2). Two millilitres of blood was submitted for haematology and biochemistry profile. The residual blood was mixed with RPMI (Gibco) containing penicillin-streptomycin (200 units/ml and 0.2 mg/ml respectively) in a 1∶2 ratio and 1 ml plated in a 24 well plate. 111.1 µl of respective ligand was added to each well (1 µg/ml rFliC and PBS control) and incubated at 37°C at 5% CO_2_ for 24 hours. The plate was then spun at 2000× g for 4 min and the supernatant harvested and stored at −80°C until analysed. The Quantikine Canine TNFα Immunoassay (R&D Systems) was used to measure TNFα production in the supernatants according to manufacturer's instruction. The OD was measured at 450 nm with a 610 nm wavelength correction using a Bio-Tek EL8088 Channel Absorbance Reader within 30 minutes and the data were analysed using Gen5 Data Analysis Software. All assays were performed in duplicate.

### Statistical analysis

For the NF-κB stimulation assays, data from four independent experiments consisting of three repeats each were pooled and presented either as relative luciferase units (RLU) or converted into fold-increase by comparing with RLU values obtained for unstimulated cells with media [37]. Pooled results were analysed using the student's t-test to determine significant differences between means of RLU between the risk-protective and risk-associated TLR5 haplotype cells for the different ligands and concentrations used in the NF-κB stimulation assays. A Mann-Whitney U test was used to determine significant differences between medians of CXCL8 concentrations between the risk-protective and risk-associated TLR5 cells for the different concentrations of flagellin used. Similarly, the Mann-Whitney U test was used to determine significant differences between medians of TNFα concentrations in GSDs carrying the risk-protective and risk-associated TLR5 polymorphisms for the different ligands used in the whole blood stimulation assay. The Mann-Whitney U test was also used to determine significant differences between medians of leucocyte subsets (neutrophils, lymphocytes, monocytes and eosinophils) in GSDs carrying the risk-protective and risk-associated TLR5 polymorphisms used in the whole blood stimulation assay. Both, Student's t-test and Mann-Whitney U test were carried out using Statistical Package for the Social Sciences (SPSS) version 15.0 software. Statistical significance was set at P<0.05. Graphs were constructed using GraphPad Prism 5 software.
